# Optimal timing of antiviral therapy for patients with malignant tumor who presented with hepatitis B reactivation during chemotherapy and/or immunosuppressive therapy

**DOI:** 10.7150/jca.40154

**Published:** 2020-03-15

**Authors:** Xiaofang Zou, Longhua Guo, Yinfang Gu, Zhijun Yang, Ping Huang, Tianhuang Liu, Jingjing Zhao, Guowu Wu

**Affiliations:** 1 Department of Medical Oncology, Cancer Center, Meizhou People's Hospital (Huangtang Hospital), Meizhou Academy of Medical Sciences, Meizhou Hospital Affiliated to Sun Yat-sen University, Meizhou, China; 2 Guangdong Provincial Key Laboratory of Precision Medicine and Clinical Translational Research of Hakka Population, Meizhou, China; 3 Department of Hepatopathy, Meizhou People's Hospital (Huangtang Hospital), Meizhou Academy of Medical Sciences, Meizhou Hospital Affiliated to Sun Yat-sen University, Meizhou, China; 4 Sun Yat-sen University Cancer Center, State Key Laboratory of Oncology in South China, Collaborative Innovation Center for Cancer Medicine, Guangzhou, China; 5 Department of Biotherapy, Sun Yat-sen University Cancer Center, Guangzhou, China

**Keywords:** antiviral prophylaxis, hepatitis B surface antigen (HBsAg)-positive, hepatitis B virus reactivation, risk factors, chemotherapy and/or immunosuppressive therapy

## Abstract

**Background**: Hepatitis B virus (HBV) reactivation may occur with chemotherapy and/or immunotherapy. Antiviral prophylaxis is recommended for all patients who are hepatitis B surface antigen (HBsAg)-positive during chemotherapy and/or immunosuppressive therapy. However, the optimal timing of antiviral therapy before chemotherapy and/or immunosuppressive therapy is not fully elucidated.

**Patients and methods**: We retrospectively evaluated 446 HBsAg-positive patients who underwent chemotherapy and/or immunosuppressive therapy. The cumulative rates of HBV reactivation were evaluated using the Kaplan-Meier method and were compared using the log-rank test. The risk factors of HBV reactivation were examined via univariate and multivariate analyses using the Cox proportional hazards model.

**Results**: The cumulative HBV reactivation rates of patients who received antiviral therapy before chemotherapy and/or immunosuppressive therapy were significantly lower than those of patients who received antiviral therapy after chemotherapy and/or immunosuppressive therapy (*P* = 0.002). The incidence of HBV reactivation was significantly different between patients who received antiviral therapy at least 1 day before chemotherapy and/or immunosuppressive therapy and those who did not (*P* = 0.006). No significant difference was observed in the HBV reactivation rates between patients who received antiviral therapy at least 2 days (*P* = 0.310), 3 days (*P* = 0.494), and 1 week (*P* = 0.655) before chemotherapy and/or immunosuppressive therapy and those who did not. The multivariate Cox proportional hazards model showed that women had a lower risk of developing HBV reactivation than men (*P* = 0.025). The use of the prophylactic antiviral agent entecavir, compared with lamivudine and telbivudine, was associated with the decreased risk of developing HBV reactivation (*P* = 0.002).

**Conclusion**: HBsAg-positive patients who received preemptive antiviral therapy after chemotherapy and/or immunosuppressive therapy had a high risk of developing HBV reactivation. However, it is not necessary for patients to receive antiviral therapy at least 1 week before chemotherapy and/or immunosuppressive therapy.

## Introduction

Hepatitis B virus (HBV) infection is prevalent worldwide 1,2. Chemotherapy and immunosuppressive therapy may reactivate such infection, with possible fatal outcomes 3. Approximately 40% of patients who are hepatitis B surface antigen (HBsAg)- positive and received chemotherapy developed HBV reactivation; among these patients, 13% and 16% are at risk of liver failure and mortality 4, respectively. Based on cautious prospective serological testing, liver damage due to HBV reactivation is a two-stage process. Initially, during intense cytotoxic or immunosuppressive therapy, a remarkedly enhanced viral replication is observed, as reflected by increases in the serum levels of HBV DNA, hepatitis B e-antigen (HBeAg), and HBV DNA polymerase, resulting in the widespread infection of hepatocytes. Due to the withdrawal of cytotoxic or immunosuppressive therapy, immune function will be restored; then, there will be a rapid immune-mediated destruction of HBV- infected hepatocytes. This destruction can manifest as hepatitis, hepatic failure, and even death 5-7.

Because hepatitis is related to HBV virological reactivation, the Centers for Disease Control and Prevention, American Association for the Study of Liver Diseases, Asian Pacific Association for the Study of the Liver, European Association for the Study of the Liver, and American Gastroenterological Association endorsed a policy involving screening for HBsAg and hepatitis B core antibody (anti-HBc) levels in patients undergoing chemotherapy or immunosuppressive therapy 2,8-11. Prophylactic antiviral therapy is recommended for all patients who are HBsAg-positive and for selected patients who are HBsAg-negative and anti-HBc-positive who receive B-cell-depleting agents or other highly aggressive chemotherapy. Therapy must be continued at least 6-12 months after the discontinuation of chemotherapy and immunosuppressive therapy. However, to date, there is no available consensus on the optimal time to initiate prophylactic antiviral agents in these conditions. Some randomized clinical trials have shown that antiviral therapy administered at least 1 week or at the start of chemotherapy or immunosuppressive therapy is more effective than deferred treatment after reactivation is diagnosed using frequent HBV DNA monitoring 2. But there is no study has shown how long to take antiviral agents in advance was preferable (one day or one week?). The intake of prophylactic antiviral agents at least 1 week before chemotherapy or immunosuppressive therapy may result in a delay of antitumor therapy and may produce additional medical costs.

Thus, we aimed to retrospectively compare the efficacy of antiviral prophylaxis before and after chemotherapy and/or immunosuppressive therapy to prevent HBV reactivation and to confirm the optimal timing of antiviral therapy for patients with malignant tumor who presented with hepatitis B virus during chemotherapy and/or immunosuppressive therapy.

## Patients and Methods

### Inclusion and exclusion criteria

The records of patients with HBV infection who received chemotherapy and/or immunosuppressive therapy between January 2014 and February 2018 in Meizhou People's Hospital (Huangtang Hospital), Meizhou Hospital Affiliated to Sun Yat-sen University in southern China were screened for eligibility. The inclusion criteria were as follows: a) patients aged ≥16 years, b) with HBsAg-positive status upon diagnosis, and c) receiving at least one cycle of chemotherapy and/or immunosuppressive therapy. The exclusion criteria were as follows: a) patients with hepatitis A, C, D, and E virus infection or HIV infection, b) with decompensated liver disease, such as a history of ascites, variceal hemorrhage, hepatic encephalopathy, or serum total bilirubin >2.0 mg/dL before chemotherapy or immunosuppressive therapy, and c) with hepatocellular carcinoma.

The institutional review board of Meizhou People's Hospital approved the study, and a written informed consent was obtained from all patients.

### Definition of HBV reactivation

HBV reactivation was defined as follows: HBV DNA level ≥20,000 IU/mL with no baseline HBV DNA, newly detected HBV DNA level ≥100 IU/mL with previously stable or undetectable levels, elevated HBV DNA ≥2 log10 with detectable HBV DNA at baseline, and reverse seroconversion to HBsAg- positive status 2,12.

### Characteristics of the patients and follow-up

The following variables were assessed: age; sex; clinical characteristics, including diagnosis of a hematologic or solid tumor; and initiation time of prophylactic antiviral and antiviral agents. Biochemical liver function tests including glutamic- pyruvic transaminase (ALT), glutamic-oxalacetic transaminase (AST), bilirubin, and albumin levels as well as HBV DNA levels were checked at baseline, at the start of every new cycle of chemotherapy and/or immunosuppressive therapy. After the completion of therapy, biochemical liver function and HBV-DNA levels were checked every 4-12 weeks. Tests for serum HBsAg, hepatitis B surface antibody (anti-HBs), HBeAg, anti-HBc status, and hepatitis B e-antibody (anti-HBe) were usually done at baseline. The follow up data was collected from January 2014 and September 2018.

### Statistical analysis

χ^2^ or Fisher's exact test was used to assess the relationship between HBV reactivation and clinicopathological features. Continuous variables without a normal distribution were compared using the Mann-Whitney U-test. The risk factors of HBV reactivation were examined via univariate and multivariate analyses using the Cox proportional hazards model. The cumulative rates of HBV reactivation were evaluated using the Kaplan-Meier method and compared using log-rank tests. A P value <0.05 for all two-tailed tests were considered statistically significant. All statistical analyses were carried out using the Statistical Package for the Social Sciences software version 19.0 (SPSS Inc., Chicago, Illinois, the USA).

## Results

### Characteristics of the patients

We identified 446 patients with resolved HBV infection who received chemotherapy or immunosuppressive therapy in the analysis. The characteristics of the patients are shown in detail in Table 1. Of the 446 patients, 239 (53.6%) were men. The median age of the participants was 52 (interquartile range [IQR]: 44-59) years. Most patients (n=391; 87.6%;) had solid tumors, and 12.4% (n=55) presented with hematological malignancies, such as leukemia or lymphoma. Among the patients with solid tumors, the most prevalent cancer site was the gastrointestinal tract (n=153; 34.3%). Seventeen (3.9%) and 75 (16.8%) patients received rituximab-based treatment regimens and anthracycline, respectively.

In terms of viral factors, 215 (48.2%) and 91 (20.4%) patients had baseline HBV DNA levels <2,000 IU/mL and <100 IU/mL, respectively, and 36 (8.1%) patients were positive for HBeAg. Most patients were treated with entecavir (ETV) (n=304; 68.2%), lamivudine (LAM) (n=117; 26.2%), or telbivudine (LDT) (n=25; 5.6%) for HBV prophylaxis. The median duration of antiviral prophylaxis before chemotherapy or immunosuppressive therapy was 1 (IQR: -1-2) day. The median duration of follow-up was 8 (IQR: 4.5-13) months. Among these patients, 52 (11.6%) were diagnosed with HBV reactivation during or after chemotherapy and/ or immunosuppressive therapy courses.

### Comparison of HBV reactivation rates between the groups according to time of treatment initiation (before or after chemotherapy and/or immunosuppressive therapy)

**Figure 1:**
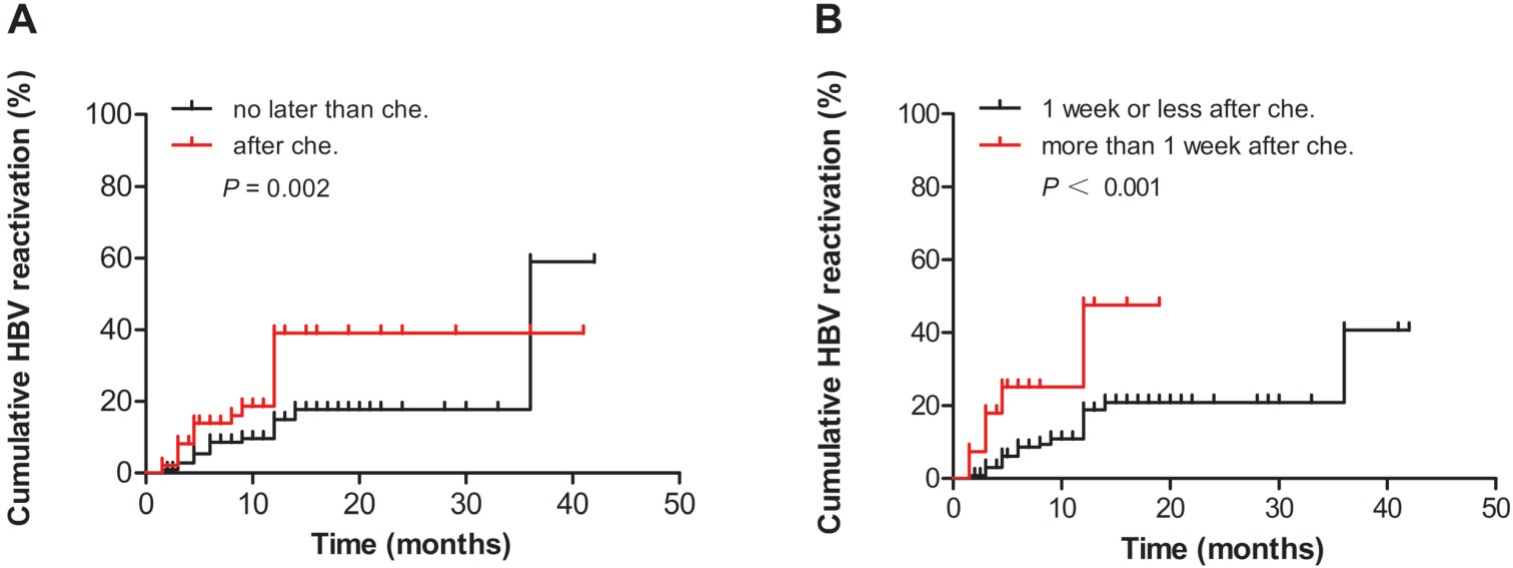
** (A)** Comparison of the cumulative HBV reactivation rates between the groups who received antiviral therapy before and after chemotherapy and/or immunosuppressive therapy. **(B)** Comparison of the cumulative HBV reactivation rates between the groups according to the time when the patients received antiviral therapy (more than or less than 1 week after chemotherapy and/or immunosuppressive therapy). Che., chemotherapy and/or immunosuppressive therapy.

We divided the patients into two groups according to the time when antiviral therapy was provided (before or after chemotherapy and/or immunosuppressive therapy). The HBV reactivation analysis showed that the cumulative HBV reactivation rates of patients who received antiviral therapy before chemotherapy and/or immunosuppressive therapy was significantly lower than those of patients who received antiviral therapy after chemotherapy and/or immunosuppressive therapy, as determined using the log-rank test (*P* = 0.002, Fig. 1A). Furthermore, those who received antiviral therapy more than 1 week after chemotherapy or immunosuppressive therapy had a significantly higher risk of HBV reactivation (*P* <0.001, Fig. 1B).

### Comparison of HBV reactivation rates between the groups according to the different durations of antiviral therapy before chemotherapy and/or immunosuppressive therapy

**Figure 2:**
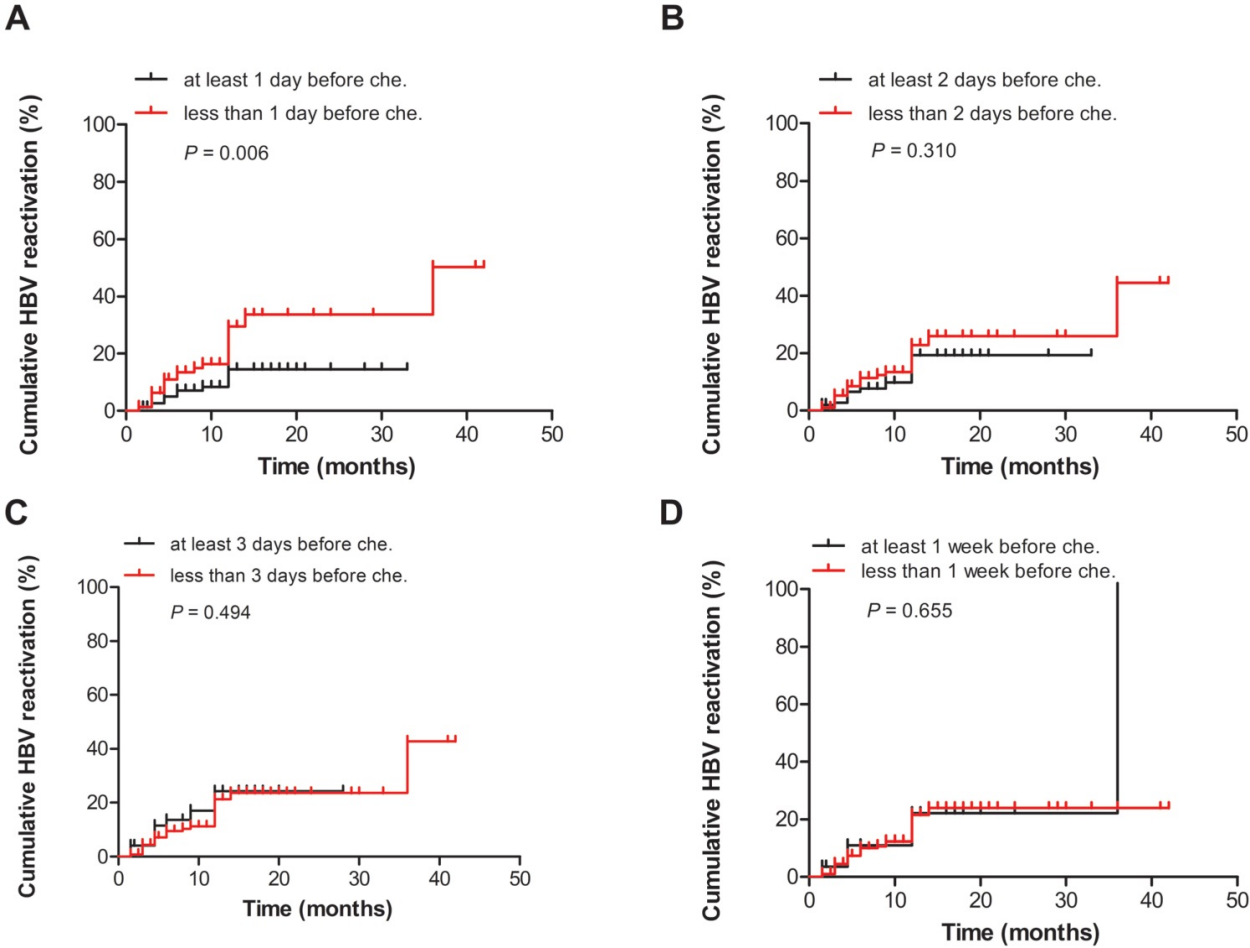
Comparison of the cumulative HBV reactivation rates between the groups according to the different durations of antiviral therapy before chemotherapy and/or immunosuppressive therapy: **(A)** at least 1 day or less than 1 day before chemotherapy and/or immunosuppressive therapy, **(B)** at least 2 days or less than 2 days before chemotherapy and/or immunosuppressive therapy, **(C)** at least 3 days or less than 3 days before chemotherapy and/or immunosuppressive therapy, and **(D)** at least 1 week or less than 1 week before chemotherapy and/or immunosuppressive therapy. Che., chemotherapy and/or immunosuppressive therapy.

The patients were divided into two groups according to the different durations of antiviral prophylaxis before chemotherapy and/or immunosuppressive therapy. The incidence of HBV reactivation was significantly different between the patients who received antiviral therapy at least 1 day before chemotherapy or immunosuppressive therapy, and patients who received antiviral therapy less than 1 day before chemotherapy or immunosuppressive therapy (*P* = 0.006, Fig. 2A). However, no significant difference was observed in the HBV reactivation rates between the two groups (at least 2 days before or less than 2 days before, *P* = 0.310; at least 3 days before or less than 3 days before, *P* = 0.494; and at least 1 week before or less than 1 week before, *P* = 0.655) (Figs. 2B, C, D).

### Clinicopathological characteristics associated with HBV reactivation

**Figure 3:**
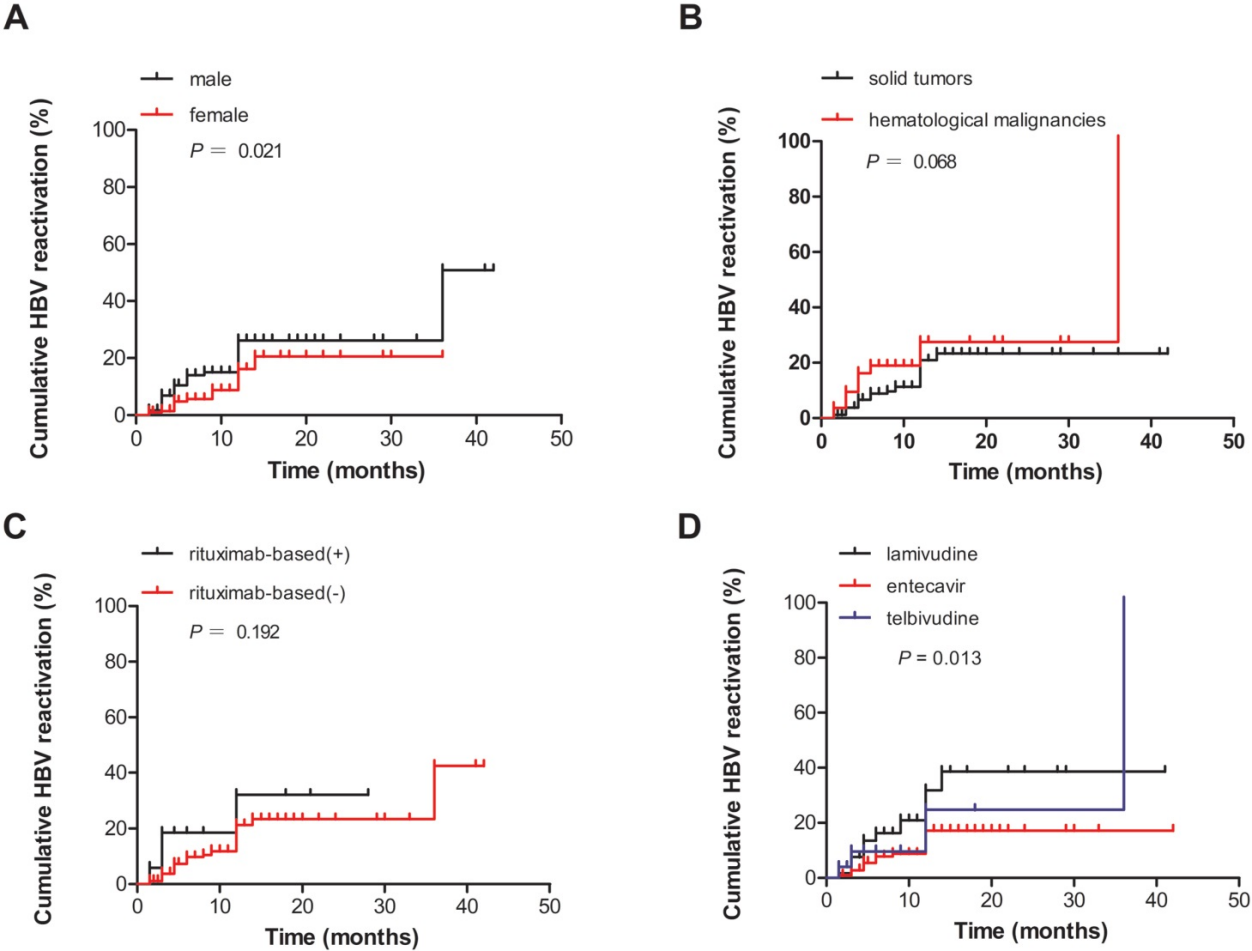
** (A)** Comparison of the cumulative HBV reactivation rates according to sex. **(B)** Comparison of the cumulative HBV reactivation rates according to different tumor types. **(C)** Comparison of the cumulative HBV reactivation rates according to the risk factors associated with rituximab-based chemotherapy or other types of treatment. **(D)** Comparison of the cumulative HBV reactivation rates according to different prophylactic antiviral regimens. Che., chemotherapy and/or immunosuppressive therapy.

A total of 52 patients developed HBV reactivation (the reactivation group), whereas 394 patients did not (the non-reactivation group). The clinical characteristics of these two groups are summarized in Table 2. The correlation analysis revealed a significant inverse correlation between the development of HBV reactivation as well as sex (male, *P* = 0.016), HBV DNA level ≥100 IU/mL (*P* = 0.048), hematological malignancies (*P* = 0.012), use of the prophylactic antiviral regimen LAM (*P* = 0.014), and administration of anti-viral prophylaxis after chemotherapy (*P* = 0.015). No statistically significant difference was observed in terms of age, baseline liver function (ALT level and HBeAg status), and chemotherapy regimens between the two groups. In addition, the cumulative HBV reactivation rates in men and those who used LAM were significantly higher than those of women and those who used ETV and LDT (*P* = 0.021 and *P* = 0.013, respectively, based on log-rank test; Figs. 3A, D). However, no significant difference was observed in the cumulative HBV reactivation rates between the groups with solid tumors and hematological malignancies and between the groups treated with rituximab and those who were not (*P* = 0.068 and *P* = 0.192, respectively, based on log-rank test; Figs. 3B, C).

Using the covariates listed in Table 3, the multivariate Cox proportional hazards model showed that men were more significantly at risk of HBV reactivation than women (HR = 0.502; 95% confidence interval (CI) = 0.276-0.915, *P* = 0.025). ETV, not LAM and LDT, was associated with a decreased risk of HBV reactivation (HR = 0.417; 95% CI = 0.238-0.731, *P* = 0.002). The administration of anti-viral prophylaxis less than 1 day before chemotherapy or immunosuppressive therapy was associated with an increased risk of HBV reactivation, as compared to at least 1 day before chemotherapy or immunosuppressive therapy (HR = 2.366; 95% CI = 1.354-4.136, *P* = 0.003) (Table 3).

## Discussion

HBV reactivation during cytotoxic chemotherapy is an important issue in clinical settings in countries, such as China, where chronic HBV infection is endemic. HBsAg-positive patients are at high risk of HBV reactivation particularly if their HBV DNA levels are elevated 13,14, and these patients should receive anti-HBV prophylaxis prior to the initiation of immunosuppressive or cytotoxic therapy, which is supported by three randomized controlled trials of HBsAg- and anti-HBc-positive patients receiving anticancer therapy 15-17. The optimal time for the initiation of antiviral prophylaxis remains uncertain. However, when such treatment is initiated only after major biochemical abnormalities are observed, the results are not entirely satisfactory. This may not be effective in reducing liver injury 18,19 because the immunologic events causing the flare have already been activated, and viral elimination is ongoing 7. Our study addressed the question whether preemptive therapy should be initiated prior or during the initiation of chemotherapy or deferred after chemotherapy. This study showed that HBsAg-positive patients are at high risk of HBV reactivation when preemptive antiviral therapy is deferred as well as previous studies 20.

Regardless of baseline serum HBV DNA levels, antiviral prophylaxis should be administered to patients with chronic hepatitis B (CHB) before the onset of anticancer therapy or a finite course of chemotherapy or immunosuppressive therapy 21. Based on the literature, antiviral agents are provided 7 days prior to treatment. To date, no studies have assessed about the initiation of antivirals before chemotherapy and/or immunosuppressive therapy. Our study showed that the incidence of HBV reactivation was significantly different between the two groups (at least 1 day before or not) (*P* = 0.006, Fig. 2A). However, no significant difference was observed in the HBV reactivation rates between the two groups (at least 2 days before or not, *P* = 0.310; at least 3 days before or not, *P* = 0.494; and at least 1 week before or not, *P* = 0.655). This may mean antiviral treatment is should be provided 1 day prior to the start of chemotherapy and/or immunosuppressive therapy, but not necessary provided 1 week prior to the start of chemotherapy and/or immunosuppressive therapy. There is no need to delay chemotherapy and/or immunosuppressive therapy administered to HBsAg-positive patients, which may lead to disease progression in order to antiviral treatment 1 week in advance.

Several risk factors for HBV reactivation in patients with cancer have been identified. According to previous studies, those with detectable or high levels of serum HBV DNA prior to the start of immunosuppressive therapy have a higher risk of HBV reactivation than those with undetectable or low levels of HBV DNA 13,14,22. In our study, a significantly lower rate of HBV reactivation was observed in patients with HBV DNA levels <100 IU/mL compared with those with HBV DNA levels ≥ 100 IU/mL. Male sex has been the most consistent host factor that is associated with an increased risk of HBV reactivation 23,24. In a study of 78 HBsAg-positive patients with various cancer types, a higher reactivation rate was observed in men than in women (29% vs 10%) 24. Our study showed that men had a higher risk of developing HBV reactivation. LAM is a nucleoside analog with potent antiviral activity against HBV and is a safe, well-tolerated, and inexpensive drug. Moreover, it is the first-line antiviral agent for the prophylaxis of HBV reactivation in patients receiving chemotherapy or immunosuppressive therapy. However, its long-term use results in drug resistance 25. Other antiviral drugs, such as ETV or tenofovir, are frequently used as alternatives to the first-line therapy for chronic hepatitis B due to the lower risk of resistance 26. A previous study has compared the prophylactic effect of LAM and ETV in patients with diffuse large B-cell lymphoma who were receiving rituximab-cyclophosphamide, doxorubicin, vincristine, and prednisone (R-CHOP) chemotherapy, and the result of such study showed that ETV was beneficial 27. In our study, a higher rate of HBV reactivation was observed in patients who used LAM as antiviral prophylaxis. Therefore, the possible relationship between HBV reactivation and the initiation time of antiviral prophylaxis in patients undergoing intense chemotherapy and/or immunotherapy must be further assessed. The present study had limitations. That is, only a short-term retrospective chart review was conducted.

## Conclusion

HBsAg-positive patients who received preemptive antiviral therapy after chemotherapy and/or immunotherapy had a high risk of developing HBV reactivation. However, it is not necessary for patients to receive antiviral therapy at least 1 week before chemotherapy. In the future, prospective studies with large sample size must be conducted to validate the optimal timing as well as the clinical and economic benefits of using antiviral prophylaxis in patients who are HBsAg-positive and are treated with chemotherapy and/or immunotherapy.

## Figures and Tables

**Table 1:** Clinical characteristics of the study participants (n=446)

Clinical characteristics	n (%)
**Sex ratio (male: female)**	239 (53.6):207 (46.4)
**Age, [median (IQR)] (years)**	52 (44-59)
**Tumor type**	
Solid tumors	391 (87.6)
Breast cancer, n (%)	80 (17.9)
Gastrointestinal cancer, n (%)	153 (34.3)
Head and neck cancer, n (%)	56 (12.5)
Lung cancer, n (%)	49 (11.0)
Genitourinary cancer, n (%)	41 (9.2)
Other cancers, n (%)	12 (2.7)
Hematological malignancies	55 (12.4)
Lymphomas, n (%)	40 (9.0)
Leukemia, n (%)	15 (3.4)
**Chemotherapy regimen**	
Anthracycline-containing	75 (16.8)
Steroid-containing	5 (1.1)
Rituximab-containing	0 (0.0)
Combined regimen with steroids and anthracycline	25 (5.6)
Combined regimen with steroids, anthracycline, and rituximab	17 (3.9)
Combined regimen with steroid and rituximab	0 (0.0)
Other chemotherapy regimen	324 (72.6)
**Duration of antiviral prophylaxis before chemotherapy (days)**	
Median (range)	1 (-1-2)
**Pretreatment (baseline) biochemistry**	
**Baseline ALT (U/L)**	26 (18-43)
≥50 U/L	91 (20.4)
<50 U/L	355 (79.6)
**Baseline HBV DNA (IU/mL)**	
≥2,000 IU/mL	231 (51.8)
<2,000 IU/mL	215 (48.2)
**Baseline HBV DNA (IU/mL)**	
≥100 IU/mL	355 (79.6)
<100 IU/mL	91 (20.4)
**Baseline ALT ≥50 U/L and HBV DNA ≥2000 IU/mL, n (%)**	50 (11.2%)
**HBeAg status**	
Positive	36 (8.1)
Negative	410 (91.9)
**HB-PreS1-Ag status**	
Positive	231 (51.8)
Negative	215 (48.2)
**Prophylactic antiviral regimen, n (%)**	
Lamivudine	117 (26.2)
Entecavir	304 (68.2)
Adefovir	0
Telbivudine	25 (5.6)
Tenofovir	0
**Duration of follow-up [median (IQR)] (months)**	8 (4.5-13)

Continuous variables expressed as median (range). ALT, alanine aminotransaminase (normal range 13-50 U/L); anti-HBe, hepatitis B e- antibody; HBeAg, hepatitis B e-antigen; HB-PreS1-Ag, hepatitis B virus PreS1-Ag; HBV, hepatitis B virus; IQR, interquartile range.

**Table 2:** Characteristics of patients with and without hepatitis B virus (HBV) reactivation

Characteristic	Patients without HBV reactivation (n =394) %	Patients with HBV reactivation (n =52) %	*P*-value
**Sex**			0.016
Male	203 (84.9)	36 (15.1)	
Female	191 (92.3)	16 (7.7)	
**Age (years)**	53 (44-59)	49 (41-57)	0.106
**ALT (U/L)**	26 (18-44)	29 (21-43)	0.384
**Elevated ALT**			0.611
Yes	79 (86.8)	12 (13.2)	
No	315 (88.7)	40 (11.3)	
**HBV DNA ≥2,000 (IU/mL)**			0.984
Yes	204 (88.3)	27 (11.7)	
No	190 (88.4)	25 (11.6)	
**HBV DNA <100 (IU/mL)**			0.048
Yes	75 (82.4)	16 (17.6)	
No	319 (89.9)	36 (10.1)	
**HBeAg**			0.212
Positive	29 (89.0)	7 (11.0)	
Negative	365 (88.3)	45 (11.7)	
**Tumor type**			0.012
Solid tumors	351 (89.8)	40 (10.2)	
**Hematological malignancies**	43 (78.2)	12 (21.8)	
**Chemotherapy regimen**			0.242
Rituximab-containing	13 (76.5)	4 (23.5)	
Without rituximab	381 (88.8)	48 (11.2)	
**Prophylactic antiviral regimen**			0.014
Lamivudine	95 (81.2)	22 (11.8)	
Entecavir	278 (91.4)	26 (8.6)	
Telbivudine	21 (84.0)	4 (16.0)	
**Antiviral prophylaxis**			0.015
Before chemotherapy	278 (90.8)	28 (9.2)	
After chemotherapy	116 (82.9)	24 (17.1)	
**Antiviral prophylaxis**			0.513
More than 1 week before chemotherapy	48 (85.7)	8 (14.3)	
One week or less before chemotherapy	346 (88.7)	44 (11.3)	

ALT, alanine aminotransaminase (normal range 13-50 U/L); HBeAg, hepatitis B e-antigen.

**Table 3:** Univariate and multivariate analyses of risk factors for HBV reactivation

Variables	Univariate analysis		Multivariate analysis	
	HR (95% CI)	*P*-value	HR (95% CI)	*P*-value
**Sex**	0.513 (0.285-0.926)	0.027	0.502 (0.276-0.915)	0.025
**Age**	0.995 (0.973-1.018)	0.688		
**Elevated ALT**	1.069 (0.559-2.044)	0.840		
**HBV DNA <100 (IU/mL)**	0.545 (0.302-0.986)	0.045	0.682 (0.374-1.245)	0.213
**HBeAg**	1.347 (0.605-2.998)	0.466		
**Tumor type**	1.788 (0.936-3.416)	0.079	1.938 (0.989-3.795)	0.054
**Rituximab- containing**	1.925 (0.692-5.356)	0.210		
**Prophylactic antiviral regimen**	0.457 (0.265-0.790)	0.005	0.417 (0.238-0.731)	0.002
**Antiviral prophylaxis**	2.265 (1.311-3.915)	0.003	2.366 (1.354-4.136)	0.003

ALT, alanine aminotransaminase (normal range 13-50 U/L); HBeAg, hepatitis B e- antigen; HR, hazard ratio.

## Abbreviations

ALT: glutamic-pyruvic transaminase
AST: glutamic-oxalacetic transaminase
anti-HBs: hepatitis B surface antibody
anti-HBe: hepatitis B e-antibody
anti-HBc: hepatitis B core antibody
CHB: chronic hepatitis B
ETV: entecavir
HBV: Hepatitis B virus
HBsAg: hepatitis B surface antigen
HBeAg: hepatitis B e-antigen
HB-PreS1-Ag: hepatitis B virus PreS1-Ag
HR: hazard ratio
IQR: interquartile range
LAM: lamivudine
LDT: telbivudine
R-CHOP: rituximab-cyclophosphamide, doxorubicin, vincristine, and prednisone
